# Induction of Diverse Bioactive Secondary Metabolites from the Mangrove Endophytic Fungus *Trichoderma* sp. (Strain 307) by Co-Cultivation with *Acinetobacter johnsonii* (Strain B2)

**DOI:** 10.3390/md15020035

**Published:** 2017-02-10

**Authors:** Liuhong Zhang, Shah Iram Niaz, Dilfaraz Khan, Zhen Wang, Yonghong Zhu, Haiyun Zhou, Yongcheng Lin, Jing Li, Lan Liu

**Affiliations:** 1 School of Marine Sciences, Sun Yat-Sen University, Guangzhou 510006, China; zhanglhn@mail2.sysu.edu.cn (L.Z.); shah_iram2000@yahoo.com (S.I.N.); dilfarazkhan@gu.edu.pk (D.K.); 2 Zhongshan School of Medicine, Sun Yat-Sen University, Guangzhou 510275, China; wangzlucky@163.com (Z.W.); zhuyongh@mail.sysu.edu.cn (Y.Z.); 3 Instrumental Analysis & Research Center, Sun Yat-Sen University, Guangzhou 510275, China; zhouhy@mail.sysu.edu.cn; 4 School of Chemistry and Chemical Engineering, Sun Yat-Sen University, Guangzhou 510275, China; ceslyc@mail.sysu.edu.cn; 5 South China Sea Bio-Resource Exploitation and Utilization Collaborative Innovation Center, Sun Yat-Sen University, Guangzhou 510275, China

**Keywords:** co-cultivation, mangrove endophytic fungus, sesquiterpene, lasiodiplodin, α-glucosidase inhibitor

## Abstract

Two new sesquiterpenes, microsphaeropsisin B (**1**) and C (**2**), and two new de-*O*-methyllasiodiplodins, (3*R*, 7*R*)-7-hydroxy-de-*O*-methyllasiodiplodin (**4**) and (3*R*)-5-oxo-de-*O*-methyllasiodiplodin (**5**), together with one new natural product (**6**) and twelve known compounds (**3**, **7**–**17**), were isolated from the co-cultivation of mangrove endophytic fungus *Trichoderma* sp. 307 and aquatic pathogenic bacterium *Acinetobacter johnsonii* B2. Their structures, including absolute configurations, were elucidated by extensive analysis of spectroscopic data, electronic circular dichroism, Mo_2_(AcO)_4_-induced circular dichroism, and comparison with reported data. All of the isolated compounds were tested for their α-glucosidase inhibitory activity and cytotoxicity. New compounds **4** and **5** exhibited potent α-glucosidase inhibitory activity with IC_50_ values of 25.8 and 54.6 µM, respectively, which were more potent than the positive control (acarbose, IC_50_ = 703.8 µM). The good results of the tested bioactivity allowed us to explore α-glucosidase inhibitors in lasiodiplodins.

## 1. Introduction

Seven examples of marine bioactive compounds or derivatives were approved by the U.S. Food and Drug Administration or in clinical trials, such as salinosporamide A, plitidepsin, bryostatin 1, cytarabine, vidarabine, eribulin mesylate, and trabectidin (ET-743) [1]. Microorganisms from the mangrove environment produce a multitude of novel and biologically-active natural products [2,3]. According to genomic studies, numerous microorganisms have far greater potential to produce specialized metabolites than was thought from classic bioactivity screens. However, dozens of fungal gene clusters may be silent under standard laboratory growth conditions, which lead to the fact that some secondary metabolites pathways cannot be expressed. Therefore, certain groups of fungi have the potential to produce even more structurally-diverse secondary metabolites if the fungal cryptic biosynthetic pathways are activated [4]. Similar studies were reported for the genomes of filamentous fungi, such as *Aspergillus* spp. [5]. Microbial interspecies competition can have dramatic effects on small molecules, which were produced to defend the habitat or as chemical signals, and may be different from their single-species counterparts [6,7]. Consequently, microorganism co-culture, which is the cultivation of two or more microorganisms in one culture vessel, and a potent way to activate the silent gene clusters and enhance chemical diversity for drug discovery, has aroused great concern in natural product research [8]. A variety of studies have explored the induction of fungal metabolites in fungal and bacterial co-cultures [9,10,11,12], as well as in fungal co-cultures [13,14,15,16].

**Figure 1:**
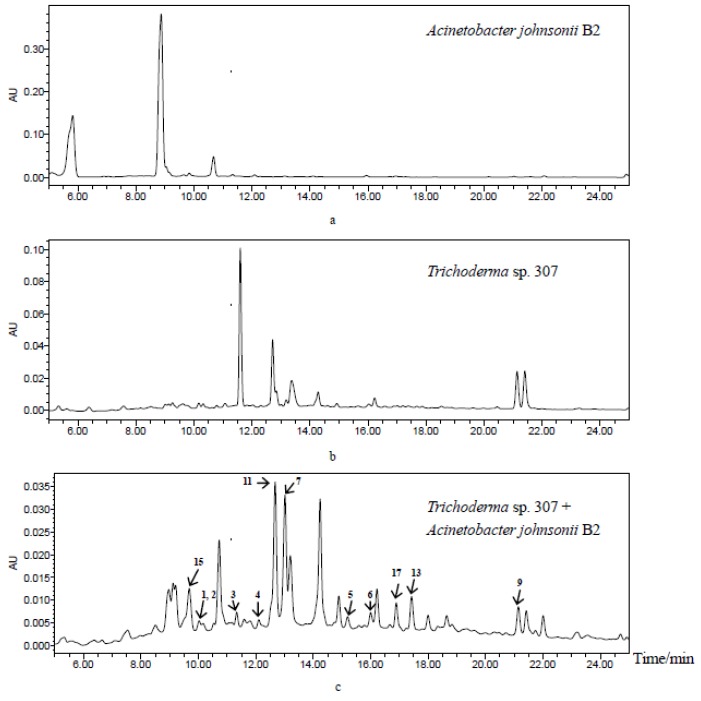
HPLC profiles of secondary metabolites from *Acinetobacter johnsonii* B2 (**a**), *Trichoderma* sp. 307 (**b**) and co-cultivation of two microorganisms (**c**) from up to down (detection wavelength: 254 nm).

In continuing the search for novel and bioactive natural products from mangrove endophytic fungi [17,18,19,20], we recently turned to our interest in microorganism co-culture in order to obtain new bioactive compounds. After analyzing the high-performance liquid chromatography (HPLC) profiles of the co-cultivation extracts of 616 strains of mangrove endophytic fungi and *Acinetobacter johnsonii* B2, together with their monoculture extracts, we found that the co-cultivation of *Trichoderma* sp. 307 and *Acinetobacter johnsonii* B2 led to the production of different metabolites to those produced in pure-cultivating of fungal and bacterial controls (Figure 1). As a result, we have discovered two new furan-type isoeremophilane sesquiterpenes (**1**–**2**), three new de-*O*-methyllasiodiplodins (**4**–**6**, including one new natural product), along with twelve known molecules (**3**, **7**–**17**). Herein, the isolation, structure elucidation, biological evaluation, and a brief discussion on the structure–activity relationship (SAR) of compounds **1**–**17** are reported.

## 2. Results and Discussion

**Figure 2:**
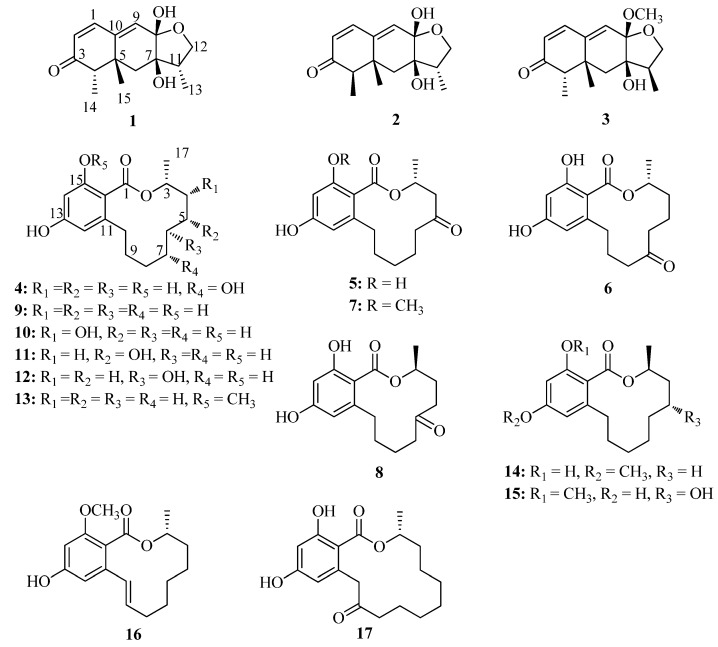
Structures of compounds **1**–**17**.

The mangrove endophytic fungus *Trichoderma* sp. 307 was co-cultured with an aquatic pathogenic bacterium named *Acinetobacter johnsonii* B2 on solid rice medium at 28 °C for 29 days. The CHCl_3_ extract of the fermentation was repeatedly fractionated and purified to obtain compounds **1**–**17** (Figure 2).

Compound **1** (1.8 mg) was obtained as a white powder. Its molecular formula C_15_H_20_O_4_ was deduced from the high resolution electrospray ionization mass spectroscopy (HRESIMS) peak at *m*/*z* 265.1438 [M + H]^+^ (calculated for C_15_H_21_O_4_, 265.1434), implying six degrees of unsaturation. The infrared radiation (IR) spectrum suggested the presence of hydroxy (3175 and 3355 cm^−1^) and conjugated carbonyl (1665 cm^−1^) groups. The ^13^C nuclear magnetic resonance (NMR) and distortionless enhancement by polarization transfer (DEPT) spectroscopic data (Table 1) revealed carbon signals for three methyl groups (δ_C_ 28.0, 14.8, and 9.2), two methylenes (δ_C_ 71.8 and 34.6), five methines (δ_C_ 146.6, 135.3, 126.3, 55.0, and 43.9), and five quaternary carbons, including one carbonyl group (δ_C_ 206.8), one ketal carbon (δ_C_ 100.5), one oxygenated carbon (δ_C_ 77.8), and two quaternary carbons (δ_C_ 139.4 and 39.9). The presence of one carbonyl group and two double bonds was attributable to three degrees of unsaturation, and the remaining three degrees of unsaturation indicated the existence of the tricyclic ring system in **1**. The ^1^H NMR and heteronuclear single-quantum correlation (HSQC) spectra of **1** (Table 1) displayed signals for three methyls [δ_H_ 1.35 (H-15, s); 1.01 (H-13, d, *J* = 7.2 Hz); 0.95 (H-14, d, *J* = 7.3 Hz)], one oxygen-bearing methylene [δ_H_ 4.07 (H-12a, dd, *J* = 8.8, 8.3 Hz); 3.34 (H-12b, dd, *J* = 8.8, 6.4 Hz)], one methylene [δ_H_ 1.79 (H-6a, d, *J* = 14.1 Hz); 1.44 (H-6b, d, *J* = 14.1 Hz)], three olefinic methines [δ_H_ 7.04 (H-1, d, *J* = 9.8 Hz); 6.10 (H-9, s); 5.84 (H-2, d, *J* = 9.8 Hz)], and two methine groups [δ_H_ 2.58 (H-11, ddq, *J* = 8.3, 7.2, 6.4 Hz); 2.24 (H-4, q, *J* = 7.3 Hz)].

**Figure 3:**
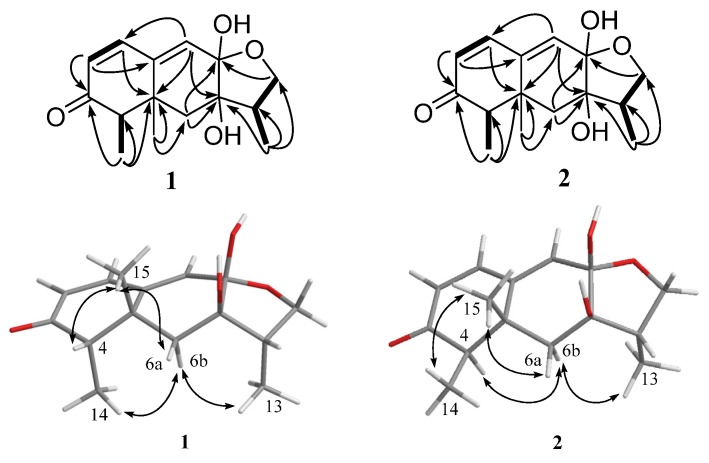
Key HMBC (black arrows), ^1^H-^1^H COSY (bold lines), and NOESY (double arrows) correlations of compounds **1** and **2**.

According to the ^1^H-^1^H correlation spectroscopy (COSY) spectrum, there were three independent spin systems of H-1/H-2, H-4/H-14, and H-12/H-11/H-13 (Figure 3). The ultra violet (UV) maximum at 284 nm revealed that the carbonyl group (δ_C_ 206.8, C-3) and the double bonds (δ_C_ 146.6, C-1; 126.3, C-2) were conjugated, which was confirmed by the heteronuclear multiple bond correlation (HMBC) correlations from olefinic protons H-1, and H-2 to C-3. The observed HMBC correlations (Figure 3) from H-1 to C-5, from H-2 to C-10, from H-6 to C-5 and C-8, from H-9 to C-1, C-5, and C-7, from H-14 to C-3, C-4, and C-5, and from H-15 to C-5, C-6, and C-10 illustrated the existence of a naphthalenone moiety with two methyl groups at C-4 and C-5, respectively. In addition, the HMBC correlations from H-11 to C-12 and C-13, H-12 to C-8, as well as H-13 to C-7, C-11, and C-12 were observed, which indicated the presence of a furan ring. As evidenced by the ^13^C NMR chemical shift (δ_C_ 100.5), C-8 was determined to be a hemiacetal carbon and was further connected to C-12 via the oxygen atom, which revealed the presence of a furan hemiacetal moiety. In the light of the NMR data (δ_C_ 77.8, C-7), the position of another hydroxyl group was assigned at C-7. Therefore, the planar structure of **1** was established.

**Figure 4:**
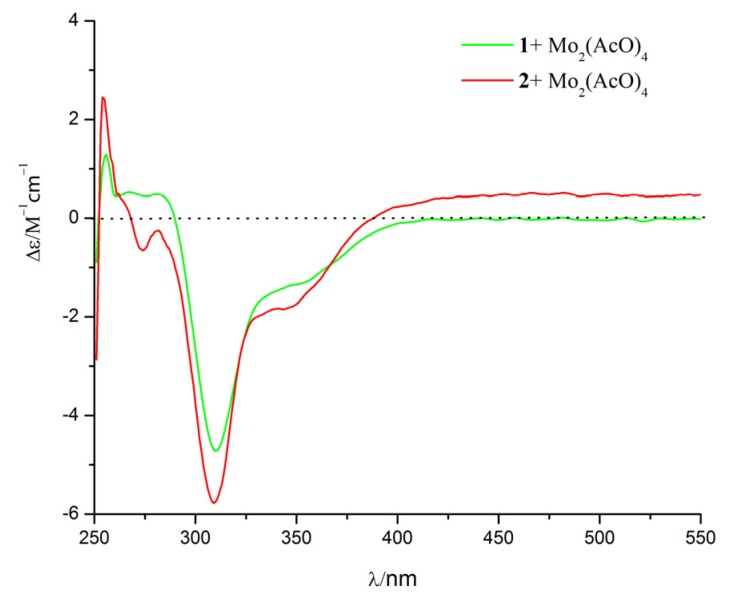
Mo_2_(AcO)_4_-induced CD spectra of compounds **1** and **2**.

**Figure 5:**
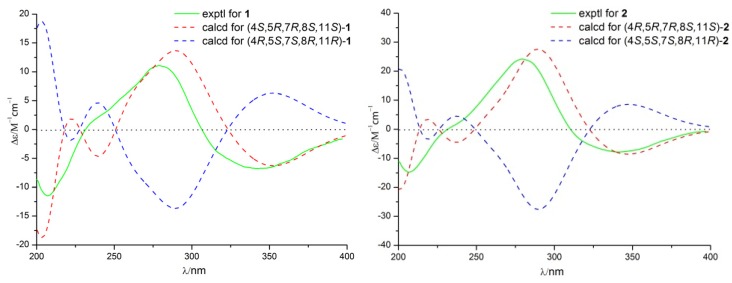
Calculated and experimental ECD spectra of compounds **1** and **2**.

The relative configuration of **1** was established by nuclear Overhauser enhancement spectroscopy (NOESY) experiment (Figure 3). The NOESY correlations of H-15 with H-4 and H-6a, and of H-6b with H-13 and H-14, revealed that H-4, H-6a, and H-15 were on the same plane of the ring system, whereas H-13 and H-14 were on the opposite side. The absolute configurations of the 7,8-diol moieties were determined by Snatzke’s method [21,22,23]. Negative Cotton effects at 310 and 400 nm in the Mo_2_(AcO)_4_-induced circular dichroism (CD) spectrum (Figure 4) suggested the 7*R* and 8*S* configurations. To support the above deduction, the theoretical electronic circular dichroism (ECD) spectrum was calculated. The calculated ECD spectrum of **1** matched well with the experimental one (Figure 5), which indicated the (4*S*, 5*R*, 7*R*, 8*S*, 11*S*)-configuration of **1**. Thus, compound **1** was a new furan-type isoeremophilane sesquiterpene, for which we suggest the trivial name microsphaeropsisin B. 

Compound **2** (3 mg) was obtained as a white powder. The molecular formula was C_15_H_20_O_4_, corresponding to six degrees of unsaturation, based on the HRESIMS peak at *m*/*z* 265.1437 [M + H]^+^ (calculated for C_15_H_21_O_4_, 265.1434). The IR spectrum indicated the presence of hydroxy (3185 and 3365 cm^−1^) and conjugated carbonyl (1670 cm^−1^) groups. The ^1^H and ^13^C NMR spectra together with HSQC correlations of **2** revealed the signals for one carbonyl group (δ_C_ 203.4), one ketal carbon (δ_C_ 100.3), one oxygenated carbon (δ_C_ 77.6), two quaternary carbons (δ_C_ 142.4 and 40.4), five methines (δ_C_ 146.1, 132.2, 128.4, 54.4 and 43.9), two methylenes (δ_C_ 71.6 and 38.9), and three methyl groups (δ_C_ 20.9, 9.2 and 7.5). A detailed comparison of the NMR data with those for compound **1** showed the presence of the same furan-type isoeremophilane sesquiterpene framework, except for the different chemical shifts of H-6, H-14, H-15, and C-6, C-10, C-14, and C-15 (Table 1). The deduction was supported by the COSY correlations from H-1 to H-2, from H-4 to H-14 and from H-11 to H-12 and H-13, along with the observed HMBC correlations from H-1 to C-3, C-5, C-9, and C-10, from H-2 to C-4 and C-10, from H-4 to C-3, C-6, C-10, and C-14, from H-6 to C-5, C-7, C-8, C-10 and C-15, from H-9 to C-1, C-5, and C-7, from H-11 to C-7, from H-12 to C-8 and C-11, from H-13 to C-7, C-11, and C-12, from H-14 to C-3, C-4, and C-5, as well as from H-15 to C-4, C-6, and C-10 (Figure 3). In addition, compound **2** and **1** had the same mass unit. Thus, we tentatively supposed that compounds **1** and **2** were a pair of epimers, which was confirmed by the increase of chemical shifts for C-6 and C-10, and the decrease of chemical shifts for C-14 and C-15, as well as the NOESY spectroscopic data analysis. The NOESY correlations of H-6a with H-15, and of H-15 with H-14 (Figure 3) suggested their *syn*-orientation, whereas the correlation of H-6b with H-4 and H-13 indicated these two protons were on the opposite face of the molecule. The absolute configurations of the 7,8-diol groups in **2** was also established by Snatzke’s method. In the Mo_2_(AcO)_4_-induced CD spectrum (Figure 4), negative Cotton effects at 309 and 398 nm supported the 7*R* and 8*S* configurations. According to a comparison of the calculated ECD spectrum with the experimental data (Figure 5), the absolute configuration of **2** was assigned as 4*R*, 5*R*, 7*R*, 8*S*, 11*S*. Therefore, the gross structure of **2** was identified as shown, named as microsphaeropsisin C.

**Figure 6:**
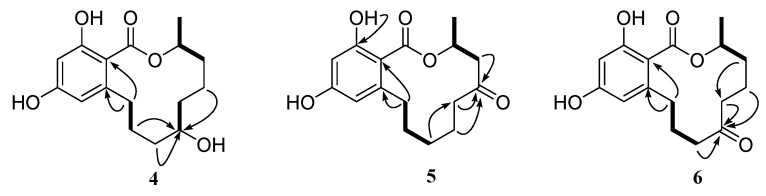
Key HMBC (black arrows) and ^1^H-^1^H COSY (bold lines) correlations of compounds **4**, **5**, and **6**.

**Figure 7:**
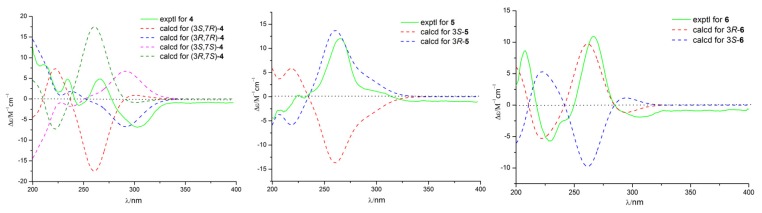
Calculated and experimental ECD spectra of compounds **4**, **5**, and **6**.

Compound **4** (2.3 mg) was obtained as a white powder and gave a molecular formula of C_16_H_22_O_5_ by HRESIMS (*m*/*z* 293.1389 [M − H]^−^, calculated for C_16_H_21_O_5_, 293.1389), with six degrees of unsaturation. The ^1^H and ^13^C NMR spectra of **4** were similar to those of de-*O*-methyllasiodiplodin [24], which suggested that compound **4** was a de-*O*-methyllasiodiplodin analogue. The major difference in the ^1^H NMR spectrum of **4** in comparison with that of de-*O*-methyllasiodiplodin was the presence of an oxygenated methine proton for H-7 that was shifted downfield to δ_H_ 4.27 (rather than one methylene protons at δ_H_ 1.42 and 1.60 in de-*O*-methyllasiodiplodin). The downfield shifts observed for C-7 in the ^13^C NMR spectrum also indicated the presence of a hydroxy group at C-7 (δ_C_ 68.0) in **4** instead of a methylene group at C-7 (δ_C_ 21.1) in de-*O*-methyllasiodiplodin. The position of the hydroxyl group at C-7 in **4** was further supported on the basis of COSY correlations from H-3 to H-4 and H-17, from H-6 to H-7 and from H-9 to H-10, along with HMBC correlations from H-4 to C-17, from H-5 to C-4, C-6 and C-17, from H-6 to C-7, from H-7 to C-6, from H-8 to C-9, and from H9 to C-6, C-7 and C-8 (Figure 6). In order to determine the absolute configuration of **4**, the theoretical ECD spectrum was calculated. As a result, the calculated curve of (3*R*, 7*R*)-**4** matched well with the experimental one (Figure 7). Hence, the structure of **4** was assigned as (3*R*, 7*R*)-7-hydroxy-de-*O*-methyllasiodiplodin. 

Compound **5** (2.3 mg) was obtained as a white powder with specific optical rotation of ${\left[\alpha \right]}_{D}^{20.0}+20.0$ (*c* 0.025, MeOH). Its molecular formula was determined as C_16_H_20_O_5_ based on HRESIMS (*m*/*z* 291.1232 [M − H]^−^, calculated for C_16_H_19_O_5_, 291.1232), with seven degrees of unsaturation. Its ^1^H NMR and ^13^C NMR data (Table 2) bore good resemblance to those of 5-oxolasiodiplodin [25], except for the presence of a chelated hydroxyl proton (δ_H_ 11.97) and the absence of the ^1^H and ^13^C signals of the methoxy group (δ_H/C_ 3.75/55.7) in **5**. Accordingly, the structure of **5** was proposed as 5-oxo-de-*O*-methyllasiodiplodin, which was confirmed by the HMBC correlations (Figure 6) from the chelated hydroxyl proton 15-OH to C-15. The absolute configuration at C-3 was determined as 3*R* by comparing the calculated ECD spectrum with the experimental one. As a result, the experimental ECD spectrum of **5** showed excellent accordance with (3*R*)-**5** (Figure 7). Thus, the structure and absolute configuration of **5** were identified as shown in Figure 1, named as (3*R*)-5-oxo-de-*O*-methyllasiodiplodin.

Compound **6** (2.6 mg) was isolated as colorless needles, and its molecular formula was determined to be C_16_H_20_O_5_ with seven degrees of unsaturation. Comparison of the 1D NMR data of **6** (Table 2) with those of **5** showed close similarity with some minor variations for the chemical shifts of C-3 through C-9, along with the change from the methylene group C-7 in de-*O*-methyllasiodiplodin to a carbonyl group at δ_C_ 211.4 in **6**. It was deduced that the position of the ketone carbonyl in the alkyl ring was changed. The deduction was supported by HMBC correlations from H-5, H-6, and H-8 to the ketone carbonyl C-7 (δ_C_ 211.4) (Figure 6). The 3*R*-configuration of **6** was determined by the comparison of the calculated ECD spectrum with the experimental one (Figure 7). To the best of our knowledge, compound **6** is reported here as a new natural product and was named as (3*R*)-7-oxo-de-*O*-methyllasiodiplodin.

The structures of compounds **3**, **7**–**17** were assigned by comparison of their spectroscopic data (1D and 2D NMR, MS) and optical rotations with published values. Therefore, the known compounds **3**, **7**–**17** were identified as microsphaeropsisin (**3**) (3.2 mg) [26], (3*R*)-5-oxolasiodiplodin (**7**) (67.9 mg) [25], (3*S*)-6-oxo-de-*O*-methyllasiodiplodin (**8**) (3.8 mg) [27], (3*R*)-de-*O*-methyllasiodiplodin (**9**) (74.3 mg) [24], (3*R*,4*R*)-4-hydroxy-de-*O*-methyllasiodiplodin (**10**) (71.8 mg) [28], (3*R*,5*R*)-5-hydroxy-de-*O*-methyllasiodiplodin (**11**) (93.1 mg) [29], (3*R*,6*R*)-6-hydroxy-de-*O*-methyllasiodiplodin (**12**) (3.5 mg) [29], (3*R*)-lasiodiplodin (**13**) (29 mg) [30], (3*S*)-ozoroalide (**14**) (15 mg) [31], (3*S*,5*R*)-5-hydroxylasiodiplodin (**15**) (16.8 mg) [25], (*E*)-9-etheno-lasiodiplodin (**16**) (15.9 mg) [27], and (3*R*)-nordinone (**17**) (18 mg) [32], respectively.

α-Glucosidase inhibitors are helpful to prevent deterioration of Type 2 diabetes and for the treatment of the disease in the early stage, which can delay the liberation of glucose from food and retard glucose absorption, thus lowering the postprandial blood glucose level [33]. Some lasiodiplodins with α-glucosidase inhibitory activity had been reported [34], so the α-glucosidase inhibitory effects of the isolated compounds were evaluated. As a result (Table 3), compounds **4**, **5**, **8**, **9**, and **10** exhibited potent α-glucosidase inhibitory activity with IC_50_ of 25.8, 54.6, 64.2, 48.9, and 60.3 µM, respectively, which were much better than acarbose (IC_50_ of 703.8 µM) as a positive control. Compounds **16** and **17** revealed seven-fold better inhibitory effects (IC_50_ of 101.3 and 105.7 μM, respectively) than acarbose. Compounds **2**, **6**, **7**, and **14** showed moderate inhibitory activity against α-glucosidase with IC_50_ values of 188.7, 178.5, 176.8, and 198.1 μM, respectively. The other molecules were inactive with IC_50_ values more than 200 μM. The results indicated that the configuration at C-5 in compounds **1** and **2** might affect α-glucosidase inhibitory activity. Moreover, the methoxy group at C-15 in the lasiodiplodin derivatives decreased the activity (**5** vs. **7** and **9** vs. **13**). For compounds **4**, **10**, **11**, and **12**, compounds **4** and **10** showed potent α-glucosidase inhibitory effects, whereas **11** and **12** were inactive, which attested that the position of the hydroxyl group had a significant impact on the activity. Similarly, according to the different activities of compounds **5**, **6**, and **8**, the position of the carbonyl moiety also exercised a great influence on the α-glucosidase inhibitory effects. In addition, the C-9-C-10 double bond of compound **16** was essential for the activity (**13** vs. **16**).

All isolates were also evaluated for their cytotoxic activity against rat pituitary adenoma GH3 cell lines and rat prolactinoma MMQ cell lines by 3-(4,5-dimethylthiazol-2-yl)-2,5-diphenyl-2*H*-tetrazolium bromide (MTT) method. Compound **9** exhibited more potent cytotoxicity against GH3 and MMQ cell lines with IC_50_ values of 6.44 and 6.58 μM, respectively, while the cytotoxicity against rat normal pituitary cells (RPC) as positive control with IC_50_ of 6.94 µM. Compound **17** displayed moderate cytotoxicity with IC_50_ values of 12.33 and 10.13 μM, respectively, which was ten-fold better than RPC cell lines with an IC_50_ value of 100.03 μM. Compound **8** was less active with IC_50_ values of 21.42 and 13.59 μM, respectively, which was seven-fold better than RPC cells with IC_50_ of 142.8 µM as positive control. However, the rest of compounds showed no cytotoxicity against the two cell lines with IC_50_ values more than 50 μM. The above consequences revealed that methylation of 13-OH or 15-OH in lasiodiplodins resulted in diminished cytotoxicity and some compounds had selective activity against rat normal cells and cancer cells. Moreover, the position of the carbonyl group and hydroxyl moiety might play a significant role in the cytotoxicity. The SAR analysis was also confirmed by our previous studies [35,36].

## 3. Materials and Methods

### 3.1. General Experimental Procedures

Optical rotations were recorded using MCP 200 Polarimeter (Anton Paar GmbH, Graz, Austria). Optical density (OD) values were read on a Multiskan Spectrum Microplate Reader (Thermo Scientific Inc., Shanghai, China). CD spectra were acquired on a Chirascan Spectrometer (Applied Photophysics Ltd., Surrey, UK). IR spectra were carried out on a Nicolet Nexus 670 spectrophotometer, in KBr discs. NMR spectra were obtained on a Bruker AVANCE 400 (Bruker Co. Ltd., Zurich, Switzerland). Thin-layer chromatography (TLC) was carried out on pre-coated silica gel GF-254 plates (Qingdao Haiyang Chemical Co., Ltd., Qingdao, China) and column chromatography (CC) was performed over silica gel (Qingdao Haiyang Chemical Co., Ltd., Qingdao, China, 200–300 mesh) on a Sephadex LH-20 (GE healthcare, Buckinghamshire, UK). Semi-preparative HPLC was performed on a Waters 1525 system using a semi-preparative Ultimate XB-C18 column (5 μm, 21.2 mm × 250 mm; Welch) coupled with a Waters 2998 photodiode array detector (Waters Corp., Milford, MA, USA). ESIMS data were measured on a Thermo LCQ DECA XP plus mass spectrometer (Thermo Scientific, Waltham, MA, USA). All reagents and solvents were of commercial quality.

### 3.2. Fungal and Bacterial Material

Strain 307, identified as *Trichoderma* sp. (GenBank accession number: KX816009), was isolated from the stem bark of *Clerodendrum inerme*, collected in Zhanjiang Mangrove National Nature Reserve in Guangdong Province, China. A voucher specimen (registration number: 307) has been deposited at the Institute of Marine Biological Natural Products, School of Marine Sciences, Sun Yat-sen University, China.

Bacterium B2, identified as *Acinetobacter johnsonii* (GenBank accession number: KY077679), was isolated from an aquaculture pond at the Maoming Experimental Station in Guangdong (Guanli Marine Organisms LLC.). A voucher specimen (registration number: B2) has been deposited at the Institute of Marine Biological Natural Products, School of Marine Sciences, Sun Yat-sen University, China.

### 3.3. Co-Cultivation, Extraction, and Isolation

Strain 307 was cultured for one week at 28 °C in five Petri dishes (i.d. 90 mm) containing 25 mL of potato dextrose agar medium. In order to obtain the mycelial suspension, the agar-supporting mycelia were cut and transferred to two 1000 mL Erlenmeyer flasks containing 500 mL of potato dextrose broth and then incubated at 28 °C for four days on a rotary shaker at 150 rpm. The bacterium B2 was cultured in a 1000 mL Erlenmeyer flask containing 500 mL of lysogeny broth at 37 °C for 24 h on a rotary shaker at 150 rpm. Then, 5 mL of the fungal seed broth and 1 mL of the bacterial seed broth were added into rice medium (94 bottles of 1000 mL Erlenmeyer flasks, each containing 50 g of rice, 100 mL distilled water), and incubated at 28 °C for 28 days under static conditions and daylight. Following incubation, the mycelia and solid rice medium were extracted three times with MeOH. The MeOH solution was concentrated under reduced pressure to afford the MeOH solution, which was extracted three times with CHCl_3_ to give 42.6 g of crude extract. The extract was then separated into 11 fractions (Fr. 1–Fr. 11) by column chromatography over silica gel eluted by a gradient of petroleum ether/EtOAc from 100:0 to 0:100 and EtOAc/MeOH from 100:0 to 0:100. Fr. 2 (319.4 mg) was applied to Sephadex LH-20 (CH_2_Cl_2_/MeOH *v*/*v*, 1:1) to yield compound **9** (74.3 mg). Fr. 3 (1355.1 mg) was divided into six fractions (Fr. 3.1 to Fr. 3.6) by CC over silica gel eluting with CHCl_3_/MeOH (*v*/*v*, 99:1), and afforded compound **14** (15 mg). Fr. 3.2 (232.1 mg) was further purified by semipreparative HPLC with 70% MeOH-H_2_O to obtain **17** (18 mg). Fr. 3.3 (301 mg) was subsequently separated on aSephadex LH-20 CC and eluted with CH_2_Cl_2_/MeOH (*v*/*v*, 1:1) to yield **13** (29 mg). Fr. 3.4 (428.2 mg) was chromatographed on silica gel (petroleum ether/EtOAc *v*/*v*, 2:8) to give subfraction Fr. 3.4.5, which was further purified by HPLC with 60% CH_3_CN-H_2_O to afford **5** (2.3 mg), and **16** (15.9 mg). Fr. 3.5 (12 mg) was separated by HPLC with 75% MeOH-H_2_O for **8** (3.8 mg) and **6** (2.6 mg). Fr. 3.6 (203.5 mg) was fractionated on a Sephadex LH-20 CC (CH_2_Cl_2_/MeOH *v*/*v*, 1:1) and further purified using HPLC eluted with 75% MeOH-H_2_O to give **10** (71.8 mg). Fr. 4 (651 mg) was applied to the Sephadex LH-20 CC eluted with CH_2_Cl_2_/MeOH (*v*/*v*, 1:1) to give subfractions Fr. 4.1 and Fr. 4.2. Fr. 4.1 (401.8 mg) was then purified by HPLC (70% MeOH-H_2_O) to yield **7** (67.9 mg). Fr. 4.2 (160.9 mg) was further purified using HPLC with 68% MeOH-H_2_O to obtain **11** (93.1 mg). Fr. 5 (463.2 mg) was subjected to silica gel CC, eluted with petroleum ether/EtOAc (*v*/*v*, 100:0 to 0:100), to obtain subfractions Fr. 5.1-6. Fr. 5.4 (52.1 mg) was purified by HPLC with 60% MeOH-H_2_O to yield **12** (3.5 mg) and Fr. 5.4.1 (11.7 mg), which was further purified using HPLC (75% MeOH-H_2_O) to obtain **3** (3.2 mg). Fr. 5.5 (72.6 mg) was purified by repeated HPLC with 70% MeOH-H_2_O to yield **4** (2.3 mg). Fr. 6 (113.9 mg) was separated into three subfractions (Fr. 6.1-3) by silica gel CC using a stepwise gradient eluting with mixtures of petroleum ether/EtOAc (*v*/*v*, 100:0 to 0:100). Fr. 6.2 (38.9 mg) was subsequently purified by HPLC eluting with 60% MeOH-H_2_O to give **1** (1.8 mg) and **15** (16.8 mg). Fr. 6.3 (50.2 mg) was purified by HPLC (60% MeOH-H_2_O) to yield 3 mg of **2**.

Microsphaeropsisin B (**1**), white powder; ${\left[\alpha \right]}_{D}^{20.0}-16.0$ (*c* 0.100, MeOH); UV (MeOH) λ_max_ (log ε) 284 (3.69) nm; ECD (MeOH) λ_max_ (Δε) 203 (−18.7), 222 (1.9), 240 (−4.6), 290 (13.7), 352 (−6.3) nm; IR (KBr) λ_max_ 3355, 3175, 1665 cm^−^^1^; ^1^H NMR (400 MHz, MeOH-*d*_4_) and ^13^CNMR (100 MHz, MeOH-*d*_4_) see Table 1; ESIMS *m*/*z* 265.1 [M + H]^+^; HRESIMS *m*/*z* 265.1438 [M + H]^+^ (calculated for C_15_H_21_O_4_, 265.1434).

Microsphaeropsisin C (**2**), white powder; ${\left[\alpha \right]}_{D}^{20.0}-124.0$ (*c* 0.025, MeOH); UV (MeOH) λ_max_ (log ε) 283 (3.79) nm; ECD (MeOH) λ_max_ (Δε) 206 (−18.7), 225 (2.7), 240 (−0.8), 288 (21.1), 353 (−8.3) nm; IR (KBr) λ_max_ 3365, 3185, 1670 cm^−^^1^; ^1^H NMR (400 MHz, MeOH-*d*_4_) and ^13^C NMR (100 MHz, MeOH-*d*_4_) see Table 1; ESIMS *m*/*z* 263.1 [M − H]^−^; HRESIMS *m*/*z* 265.1437 [M + H]^+^ (calculated for C_15_H_21_O_4_, 265.1434).

(3*R*, 7*R*)-7-hydroxy-de-*O*-methyllasiodiplodin (**4**), white powder; ${\left[\alpha \right]}_{D}^{20.0}+6.06$ (*c* 0.033, MeOH); ECD (MeOH) λ_max_ (Δε) 228 (0.9), 238 (1.9), 292 (−6.7) nm; ^1^H NMR (400 MHz, pyridine-*d*_5_) and ^13^C NMR (100 MHz, pyridine-*d*_5_) see Table 2; ESIMS *m*/*z* 293.0 [M − H]^−^; HRESIMS *m*/*z* 293.1389 [M − H]^−^, calculated for C_16_H_21_O_5_, 293.1389).

(3*R*)-5-oxo-de-*O*-methyllasiodiplodin (**5**), white powder; ${\left[\alpha \right]}_{D}^{20.0}+20.0$ (*c* 0.025, MeOH); ECD (MeOH) λ_max_ (Δε) 207 (−3.6), 219 (−5.8), 260 (13.7) nm; ^1^H NMR (400 MHz, CDCl_3_) and ^13^C NMR (100 MHz, CDCl_3_) see Table 2; HRESIMS *m*/*z* 291.1232 [M − H]^−^, calculated for C_16_H_19_O_5_, 291.1232).

(3*R*)-7-oxo-de-*O*-methyllasiodiplodin (**6**), colorless needles; ${\left[\alpha \right]}_{D}^{20.0}+160$ (*c* 0.020, CHCl_3_); ECD (MeOH) λ_max_ (Δε) 223 (−5.3), 263 (9.7) nm; ^1^H NMR (400 MHz, CDCl_3_) and ^13^C NMR (100 MHz, CDCl_3_) see Table 2; HRESIMS *m*/*z* 291.1231 [M − H]^−^, calculated for C_16_H_19_O_5_, 291.1232).

### 3.4. Calculation of ECD Spectra

The molecular Dreiding force field was run with Spartan 14 software (Wavefunction Inc., Irvine, CA, USA). The time-dependent density functional theory (TDDFT) calculations were carried out with Gaussian 05 (Gaussian, Wallingford, CT, USA). The energy-minimized conformers were generated and optimized at the B3LYP/6-31G (d) level. The integral equation formalism variant polarizable continuum model (IEF-PCM) solvent model for MeOH was used. The ECD spectra were calculated at the RB3LYP/6-311++G (2d, p) level by SpecDis 3.0 (University of Würzburg, Würzburg, Germany) and OriginPro 8.5 (OriginLab, Ltd., Northampton, MA, USA) based on the final optimized structures. All calculations were performed by the high-performance grid computing platform at Sun Yat-Sen University.

### 3.5. α-Glucosidase Inhibitory Activity Assay

The assay of α-glucosidase inhibitory activity was carried out according to the reported method, with minor modifications [33]. All of the assays were performed under 0.01 M potassium phosphate buffer (pH 7). Enzyme solutions were prepared to give 2.0 Units/mL in 2 mL buffer solution. Test samples were dissolved in dimethyl sulphoxide (DMSO) to give an initial concentration of 4000 μM/L. One-hundred fifty-five microliters of phosphate buffer, 10 μL of test samples, and 10 μL of diluted enzyme solution were mixed in each well of a 96-well microtiter plate. After 20 min incubation at 37 °C, 25 μL of substrate (*p*-nitrophenyl-α-d-glucopyranoside, 1.5 mg/mL) was added to each well to begin the enzymatic reaction. The reaction was monitored spectrophotometrically by measuring the absorbance at 410 nm for a 1 min interval. Acarbose was used as a positive control. Calculations were performed according to the following equation: the inhibition rates (%) = [(*B* − *S*)/*B*] × 100% (*B* represents the OD value in the assay medium with DMSO, *S* represents the OD value in the assay medium with test samples or acarbose). All measurements were done in triplicate from two independent experiments. The reported IC_50_ was the average value of two independent experiments.

### 3.6. Cytotoxicity Assay

The cytotoxic activities against rat pituitary adenoma MMQ and GH3 cell lines were evaluated by MTT assay following the previous process [36,37]. Briefly, MMQ and GH3 cell lines were seeded in 96-well plates (Corning, New York, NY, USA) at a density of 5 × 10^4^ cells per well. Then, 10 μL of MTT reagent was added to each well and incubated at 37 °C with 5% CO_2_ for 4 h. Subsequently, 100 μL of acidified isopropyl alcohol was added. Then the OD value was measured at 450 nm using a microplate reader and the cell proliferation rate relative to the control was calculated. The IC_50_ was analyzed by SPSS 13.0 (SPSS, Chicago, IL, USA). Rat normal pituitary cells were used as a positive control.

### 3.7. HPLC Profiles Conditions

The detection was operated on a Waters 1525 system coupled with a Waters 2998 photodiode array detector (Waters Corp., Milford, MA, USA). The samples were eluted from an analytical Ultimate XB-C18 column (5 μm, 4.6 × 250 mm; Welch) at a flow rate of 1.0 mL/min using the following binary gradient with solvent A consisting of 15% acetonitrile/85% H_2_O and solvent B consisting of 15% H_2_O/85% acetonitrile: 0–22 min, 100%A–100%B; 22–27 min, 100%B; 27–35 min, 100%B–100%A. The detection wavelength was from 200 to 700 nm. The temperature was maintained at 25 °C, and the injection volume was 30 µL.

## 4. Conclusions

A chemical investigation of the co-cultivation of *Trichoderma* sp. 307 and *Acinetobacter johnsonii* B2 led to the isolation of two new sesquiterpenes (**1**–**2**), two new de-*O*-methyllasiodiplodins (**4**–**5**), one new natural product (**6**), along with twelve known molecules (**3**, **7**–**17**). To the best of our knowledge, compounds **1** and **2** were two unusual furan-type isoeremophilane sesquiterpenes. It is reasonable to deem that these compounds were produced by the fungus instead of the bacterium during the fungal-bacterial co-culture on the basis of their structures, their known fungal origins and their HPLC profiles. The α-glucosidase inhibitory effects and cytotoxicity of these isolated compounds were also estimated. The new compounds **4** and **5** revealed more potent inhibitory activity against α-glucosidase than the clinical α-glucosidase inhibitor acarbose, which allowed us to explore α-glucosidase inhibitors in lasiodiplodins. 

## Figures and Tables

**Table 1:** ^1^H (400 MHz) and ^13^C (100 MHz) NMR data of **1** and **2**.

Position	1 ^a^		2 ^a^	
	δ_C_	δ_H_ (*J* in Hz)	δ_C_	δ_H_ (*J* in Hz)
1	146.6	7.04 (d, *J* = 9.8)	146.1	7.01 (d, *J* = 9.8)
2	126.3	5.84 (d, *J* = 9.8)	128.4	5.93 (d, *J* = 9.8)
3	206.8		203.4	
4	55.0	2.24 (q, *J* = 7.3)	54.4	2.24 (q, *J* = 6.9)
5	39.9		40.4	
6a	34.6	1.79 (d, *J* = 14.1)	38.9	1.87 (d, *J* = 14.0)
6b		1.44 (d, *J* = 14.1)		1.51 (d, *J* = 14.0)
7	77.8		77.6	
8	100.5		100.3	
9	135.3	6.10 (s)	132.2	5.94 (s)
10	139.4		142.4	
11	43.9	2.58 (ddq, *J* = 8.3, 7.2, 6.4)	43.9	2.54 (ddq, *J* = 7.8, 6.9, 6.4)
12a	71.8	4.07 (dd, *J* = 8.8, 8.3)	71.6	4.04 (dd, *J* = 8.3, 7.8)
12b		3.34 (dd, *J* = 8.8, 6.4)		3.31 (dd, *J* = 8.3, 6.4)
13	9.2	1.01 (d, *J* = 7.2)	9.2	1.01 (d, *J* = 6.9)
14	14.8	0.95 (d, *J* = 7.3)	7.5	1.08 (d, *J* = 6.9)
15	28.0	1.35 (s)	20.9	1.16 (s)

^a^ Measured in MeOH-*d*_4_.

**Table 2:** ^1^H (400 MHz) and ^13^C (100 MHz) NMR data of **4**–**6**.

Position	4 ^a^		5 ^b^		6 ^b^	
	δ_C_	δ_H_ (*J* in Hz)	δ_C_	δ_H_ (*J* in Hz)	δ_C_	δ_H_ (*J* in Hz)
1	172.0		171.0		171.8	
3	73.7	5.37 (m)	69.3	5.69 (ddq, *J* = 10.8, 6.3, 2.3)	74.9	5.28 (ddq, *J* = 12.8, 6.3, 3.3)
4	31.4	1.94 (m)	46.1	3.31 (dd, *J* = 16.4, 10.8)	31.7	1.94 (dd, *J* = 12.8, 7.7)
		1.65 (m)		2.39 (dd, *J* = 16.4, 2.3)		1.84 (dd, *J* = 7.7, 3.3)
5	30.2	1.74 (m)	210.7		19.2	2.08 (m)
		1.28 (m)				1.76 (m)
6	32.5	2.12 (m)	46.1	2.61 (m)	38.6	2.64 (m)
		1.83 (m)		2.30 (m)		2.55 (m)
7	68.0	4.27 (m)	21.8	2.01 (m)	211.4	
				1.86 (m)		
8	24.1	1.92 (m)	29.9	1.48 (m)	42.1	2.44 (m)
		1.67 (m)		1.25 (m)		2.35 (m)
9	31.1	2.06 (m)	30.3	1.66 (m)	28.9	2.20 (m)
		1.76 (m)		1.57 (m)		1.75 (m)
10	33.7	3.22 (m)	36.8	3.45 (m)	33.6	3.14 (m)
		2.59 (m)		2.11 (m)		2.61 (m)
11	148.3		149.0		148.0	
12	111.9	6.74 (d, *J* = 2.4)	111.1	6.17 (d, *J* = 2.6)	110.8	6.24 (d, *J* = 2.6)
13	163.9		160.5		160.5	
14	102.3	6.84 (d, *J* = 2.4)	101.8	6.26 (d, *J* = 2.6)	102.0	6.30 (d, *J* = 2.6)
15	164.9		166.1		165.8	
16	107.6		104.8		106.0	
17	18.2	1.38 (d, *J* = 6.3)	19.9	1.44 (d, *J* = 6.3)	19.6	1.35 (d, *J* = 6.3)
15-OH		12.42 (s)		11.97 (s)		12.05(s)

^a^ Measured in pyridine-*d*_5_; ^b^ Measured in CDCl_3_.

**Table 3:** α-Glucosidase inhibitory activities ^a^.

Compounds	IC_50_ (µM)	Compounds	IC_50_ (µM)
**1**	>200	**10**	60.3 ± 0.7
**2**	188.7 ± 1.2	**11**	>200
**3**	>200	**12**	>200
**4**	25.8 ± 0.2	**13**	>200
**5**	54.6 ± 0.5	**14**	198.1 ± 1.5
**6**	178.5 ± 1.1	**15**	˃200
**7**	176.8 ± 1.4	**16**	101.3 ± 0.9
**8**	64.2 ± 0.5	**17**	105.7 ± 1.0
**9**	48.9 ± 0.4	Acarbose ^b^	703.8 ± 2.2

^a^ IC_50_ values are shown as mean ± SD from two independent experiments; ^b^ Positive control.

## Supplementary Materials

Supplementary materials relating to this article is available online at www.mdpi.com/1660-3397/15/2/35/s1. Figure S1: The HRESIMS spectrum of compound **1**, Figure S2: The ^13^C NMR spectrum of compound **1**, Figure S3: The ^1^H NMR spectrum of compound **1**, Figure S4: The HSQC spectrum of compound **1**, Figure S5: The ^1^H-^1^H COSY spectrum of compound **1**, Figure S6: The HMBC spectrum of compound **1**, Figure S7: The NOESY spectrum of compound **1**, Figure S8: The HRESIMS spectrum of compound **2**, Figure S9: The ^13^C NMR spectrum of compound **2**, Figure S10: The ^1^H NMR spectrum of compound **2**, Figure S11: The HSQC spectrum of compound **2**, Figure S12: The ^1^H-^1^H COSY spectrum of compound **2**, Figure S13: The HMBC spectrum of compound **2**, Figure S14: The NOESY spectrum of compound **2**, Figure S15: The HRESIMS spectrum of compound **4**, Figure S16: The ^13^C NMR spectrum of compound **4**, Figure S17: The ^1^H NMR spectrum of compound **4**, Figure S18: The HSQC spectrum of compound **4**, Figure S19: The ^1^H-^1^H COSY spectrum of compound **4**, Figure S20: The HMBC spectrum of compound **4**, Figure S21: The NOESY spectrum of compound **4**, Figure S22: The HRESIMS spectrum of compound **5**, Figure S23: The ^13^C NMR spectrum of compound **5**, Figure S24: The ^1^H NMR spectrum of compound **5**, Figure S25: The HSQC spectrum of compound **5**, Figure S26: The ^1^H-^1^H COSY spectrum of compound **5**, Figure S27: The HMBC spectrum of compound **5**, Figure S28: The NOESY spectrum of compound **5**, Figure S29: The HRESIMS spectrum of compound **6**, Figure S30: The ^13^C NMR spectrum of compound **6**, Figure S31: The ^1^H NMR spectrum of compound **6**, Figure S32: The HSQC spectrum of compound **6**, Figure S33: The ^1^H-^1^H COSY spectrum of compound **6**, Figure S34: The HMBC spectrum of compound **6**, Figure S35: The NOESY spectrum of compound **6**.

## Abbreviations

The following abbreviations are used in this manuscript:

ECD	Electronic Circular Dichroism
MTT	3-(4,5-dimethylthiazol-2-yl)-2,5-diphenyl-2*H*-tetrazolium bromide
HPLC	High Performance Liquid Chromatography
