# Supplementary material for: Induction of Diverse Bioactive Secondary Metabolites from the Mangrove Endophytic Fungus Trichoderma sp. (Strain 307) by Co-Cultivation with Acinetobacter johnsonii (Strain B2)
Source: Mar Drugs. 2017 Feb 10;15(2):35. doi: 10.3390/md15020035 (PMC5334615; doi:10.3390/md15020035)
Supplement: Supplementary file 1 [file marinedrugs-15-00035-s001.docx]

**Supplementary Materials: Induction of Diverse Bioactive Secondary Metabolites from the Mangrove Endophytic Fungus *Trichoderma* sp. (Strain 307) by Co-Cultivation with *Acinetobacter johnsonii*
(Strain B2)**

Liuhong Zhang, Shah Iram Niaz, Dilfaraz Khan, Zhen Wang, Yonghong Zhu, Haiyun Zhou, Yongcheng Lin, Jing Li and Lan Liu

Spectroscopic data of the known compounds:

Microsphaeropsisin (**3**), white solid; ^1^H NMR (400 MHz, CDCl_3_) δ_H_: 6.90, 6.11, 6.01, 4.10, 3.49, 3.01, 2.37, 2.09, 1.59, 1.20, 1.05; ^13^C NMR (100 MHz, CDCl_3_) δ_C_: 201.2, 144.0, 143.8, 143.5, 128.6, 124.4, 101.2, 78.1, 72.8, 53.0, 48.6, 46.4, 43.1, 40.4, 20.5, 14.4, 7.3; ESIMS *m*/*z* 263 [M − CH_3_]^−^.

(3*R*)-5-oxolasiodiplodin (**7**), white powder; ^1^H NMR (400 MHz, CDCl_3_) *δ*_H_: 6.23, 6.18, 5.65, 3.72, 2.88, 2.64, 2.51, 2.47, 2.47, 2.30, 1.90, 1.66, 1.50, 1.50, 1.43, 1.43, 1.36; ^13^C NMR (100 MHz, CDCl_3_) *δ*_C_: 210.2, 167.9, 158.4, 157.8, 142.9, 116.3, 108.7, 97.1, 69.0, 56.0, 49.2, 42.2, 32.7, 29.2, 27.4, 21.8, 20.4; ESIMS *m*/*z* 305 [M − H] ^−^.

(3*S*)-6-oxo-de-*O*-methyllasiodiplodin (**8**), white powder; ^1^H NMR (400 MHz, CDCl_3_) δ_H_: 11.9, 6.24, 6.17, 5.33, 3.09, 2.76, 2.72, 2.56, 2.49, 2.44, 2.23, 1.85, 1.72, 1.60, 1.48, 1.40; ^13^C NMR (100 MHz, CDCl_3_) δ_C_: 211.7, 171.7, 165.9, 160.5, 148.8, 110.7, 105.2, 101.7, 73.1, 40.3, 38.1, 34.3, 31.4, 31.0, 22.2, 19.2; ESIMS *m*/*z* 291 [M − H]^−^.

(3*R*)-de-*O*-methyllasiodiplodin (**9**), colorless needles; ^1^H NMR (400 MHz, CDCl_3_) δ_H_: 12.12, 6.29, 6.24, 5.14, 3.25, 2.47, 1.90, 1.76, 1.56-1.64, 1.60, 1.50-1.62, 1.49, 1.49, 1.42, 1.42, 1.42, 1.36; ^13^C NMR (100 MHz, CDCl_3_) δ_C_: 172.1, 165.2, 160.3, 149.6, 111.1, 105.5, 101.5, 75.4, 33.6, 31.1, 30.9, 27.3, 24.8, 24.2, 21.2, 20.2; ESIMS *m*/*z* 277 [M − H]^−^.

(3*R*,4*R*)-4-hydroxy-de-*O*-methyllasiodiplodin (**10**), white powder; ^1^H NMR (400 MHz, Acetone-*d*_6_) δ_H_: 6.31, 6.24, 4.90, 3.83, 3.52, 2.31, 1.90, 1.68, 1.67, 1.62, 1.57, 1.54, 1.46; ^13^C NMR (100 MHz, Acetone-*d*_6_) δ_C_: 172.3, 166.4, 163.5, 149.8, 112.2, 104.9, 101.9, 77.3, 71.8, 34.9, 34.6, 33.1, 29.3, 25.6, 22.7, 17.8; ESIMS *m*/*z* 293 [M − H]^−^.

(3*R*,5*R*)-5-hydroxy-de-*O*-methyllasiodiplodin (**11**), white powder; ^1^H NMR (400 MHz, Acetone-*d*_6_) δ_H_: 6.29, 6.23, 5.22, 4.01, 3.72, 2.27, 2.11, 1.69, 1.67, 1.65, 1.63–1.50, 1.46, 1.37, 1.30; ^13^C NMR (100 MHz, Acetone-*d*_6_) δ_C_: 172.8, 166.8, 163.7, 150.1, 112.2, 104.5, 102.0, 73.4, 69.8, 41.8, 35.7, 34.8, 32.7, 28.8, 21.7, 19.2; ESIMS *m*/*z* 293 [M − H]^−^.

(3*R*,6*R*)-6-hydroxy-de-*O*-methyllasiodiplodin (**12**), white powder; ^1^H NMR (400 MHz, CD_3_OD) δ_H_: 6.28, 6.22, 5.12, 4.06, 3.70, 2.20, 2.06, 1.85, 1.83, 1.70, 1.67, 1.63, 1.60, 1.44, 1.30, 1.30, 1.22; ^13^C NMR (100 MHz, CD_3_OD) δ_C_: 173.2, 166.6, 163.9, 149.7, 111.9, 105.1, 102.0, 76.9, 66.8, 37.1, 34.8, 33.1, 32.9, 31.9, 24.0, 22.0; ESIMS *m*/*z* 293 [M − H]^−^.

(3*R*)-lasiodiplodin (**13**), colorless needles; ^1^H NMR (400 MHz, CDCl_3_) δ_H_: 6.19, 6.18, 5.28, 3.67, 2.60, 2.44, 1.91, 1.67, 1.64, 1.61, 1.53, 1.49, 1.48, 1.41, 1.40, 1.32, 1.31, 1.24; ^13^C NMR (100 MHz, CDCl_3_) δ_C_: 168.7, 160.0, 158.9, 143.2, 118.1, 108.8, 97.7, 71.9, 56.1, 33.2, 30.9, 30.5, 27.4, 25.8, 25.3, 21.6, 19.8; ESIMS *m*/*z* 291 [M − H]^−^.

(3*S*)-ozoroalide (**14**), colorless needles; ^1^H NMR (400 MHz, CDCl_3_) δ_H_: 6.19, 6.18, 5.28, 3.66, 2.60, 2.44, 1.91, 1.67, 1.64, 1.61, 1.53, 1.49, 1.48, 1.41, 1.40, 1.32, 1.31, 1.24; ^13^C NMR (100 MHz, CDCl_3_) δ_C_: 169.6, 158.0, 158.0, 143.1, 117.1, 108.5, 97.1, 72.7, 55.9, 32.4, 30.5, 30.2, 26.5, 25.6, 24.2, 21.4, 19.6; ESIMS *m*/*z* 291 [M − H]^−^.

(3*S*,5*R*)-5-hydroxylasiodiplodin (**15**), white powder; ^1^H NMR (400 MHz, CD_3_OD) δ_H_: 6.31, 6.25, 5.31, 3.77, 3.76, 2.73, 2.43, 2.00, 1.79, 1.65, 1.64, 1.55, 1.54, 1.43–1.39, 1.37; ^13^C NMR (100 MHz, CD_3_OD) δ_C_: 170.7, 160.9, 159.9, 144.0, 116.7, 109.2, 97.8, 72.0, 71.6, 56.2, 44.6, 37.3, 31.9, 30.8, 27.8, 23.2, 21.9; ESIMS *m*/*z* 307 [M − H]^−^.

(*E*)-9-etheno-lasiodiplodin (**16**), white powder; ^1^H NMR (400 MHz, CDCl_3_) δ_H_: 6.40, 6.34, 6.34, 5.84, 5.22, 5.17, 3.82, 2.26, 1.86, 1.61, 1.58, 1.52, 1.48, 1.43, 1.37, 1.35, 1.33; ^13^C NMR (100 MHz, CDCl_3_) δ_C_: 168.2, 158.0, 157.5, 139.5, 135.0, 129.1, 115.9, 105.7, 97.7, 72.6, 56.1, 33.6, 31.9, 27.5, 24.9, 21.8, 20.3; ESIMS *m*/*z* 289 [M − H]^−^.

(3*R*)-nordinone (**17**), white powder; ^1^H NMR (400 MHz, Acetone-*d_6_*) δ_H_: 6.29, 6.18, 5.15, 4.60, 3.78, 2.78, 2.54, 1.85, 1.80, 1.52, 1.42, 1.35-1.32, 1.33, 1.30; ^13^C NMR (100 MHz, Acetone-*d*_6_) δ_C_: 207.0, 172.3, 166.5, 163.0, 141.0, 113.8, 102.7, 74.4, 50.5, 39.4, 35.2, 27.5, 24.6, 23.7, 23.3, 22.4, 21.3; ESIMS *m*/*z* 319 [M − H]^−^.

**Figure S1.** The HRESIMS spectrum of compound **1**.


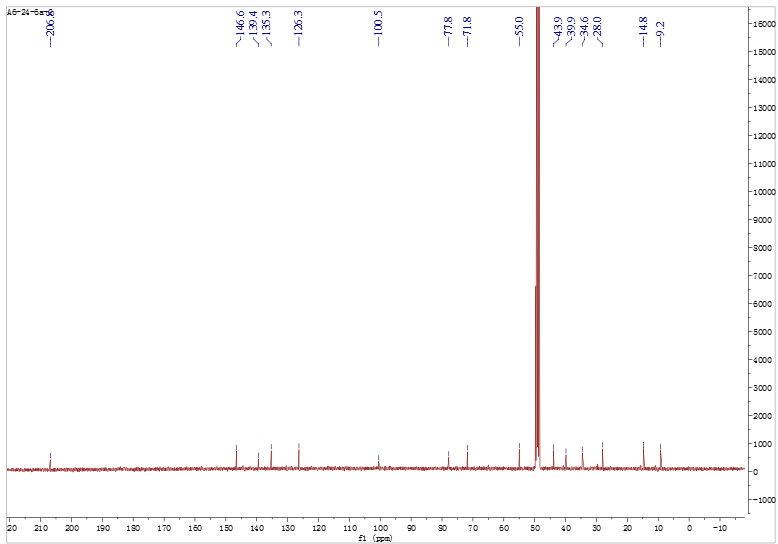


**Figure S2.** The ^13^C NMR spectrum of compound **1**.


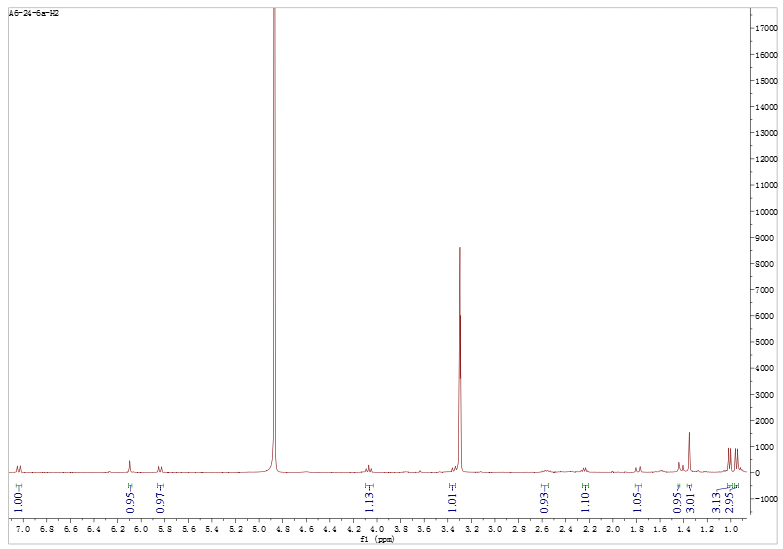


**Figure S3.** The ^1^H NMR spectrum of compound **1**.


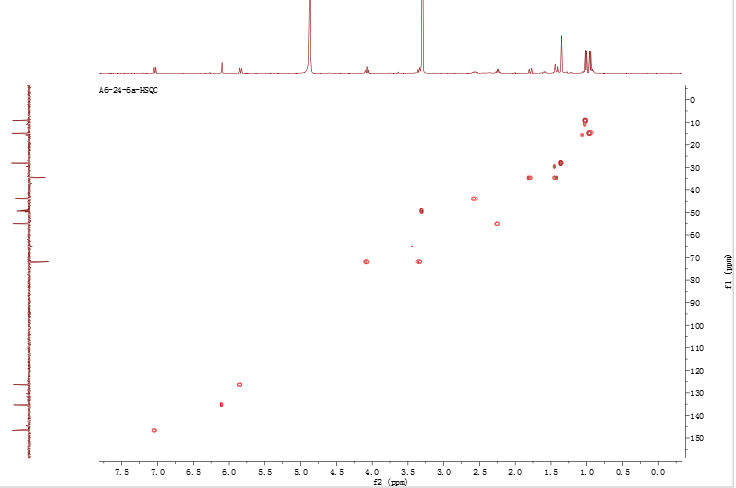


**Figure S4.** The HSQC spectrum of compound **1**.


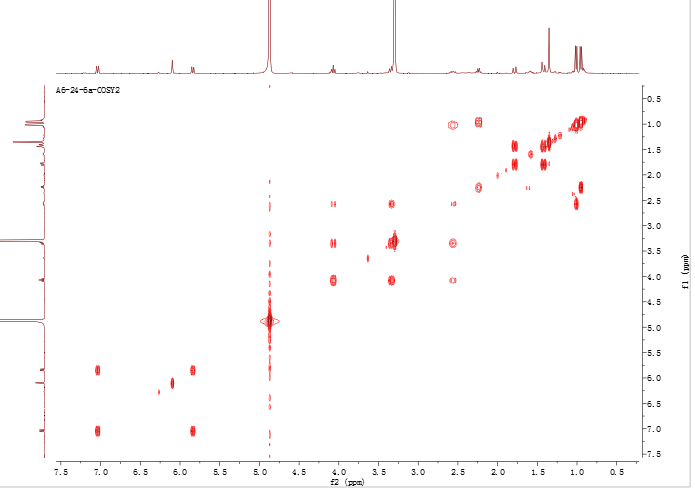


**Figure S5.** The ^1^H-^1^H COSY spectrum of compound **1**.


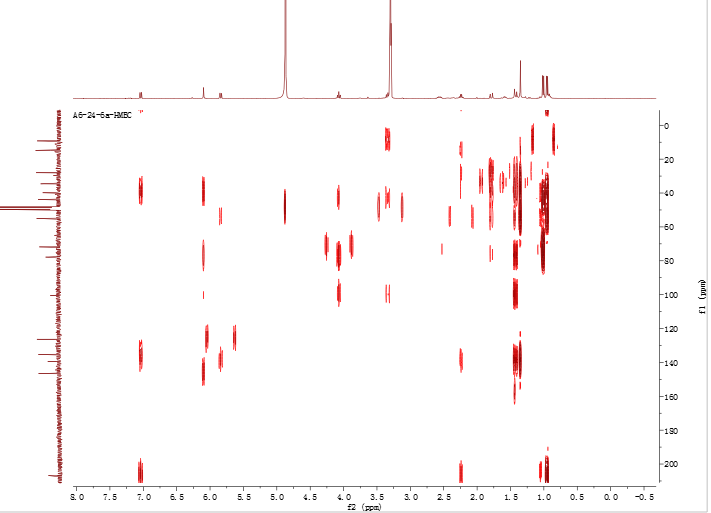


**Figure S6.** The HMBC spectrum of compound **1**.


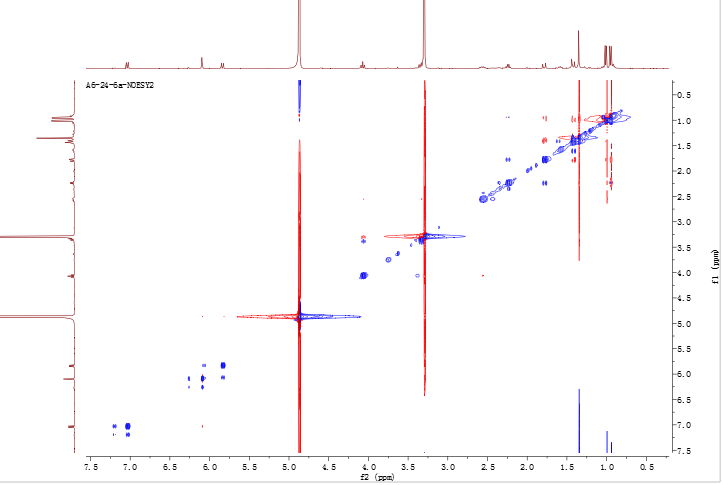


**Figure S7.** The NOESY spectrum of compound **1**.

**Figure S8.** The HRESIMS spectrum of compound **2**.


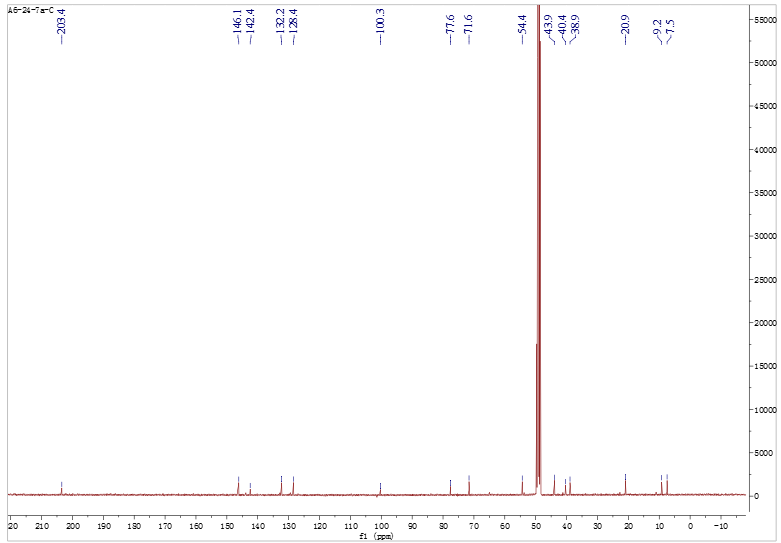


**Figure S9.** The ^13^C NMR spectrum of compound **2**.


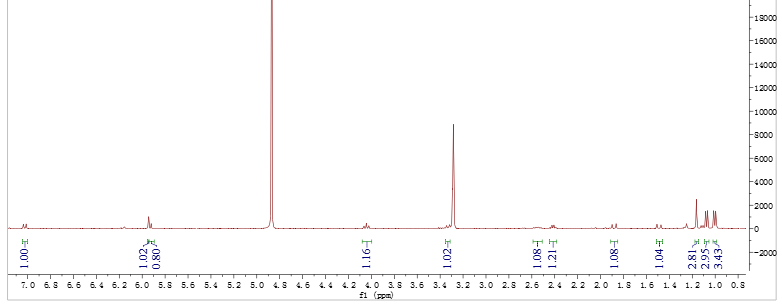


**Figure S10.** The ^1^H NMR spectrum of compound **2**.


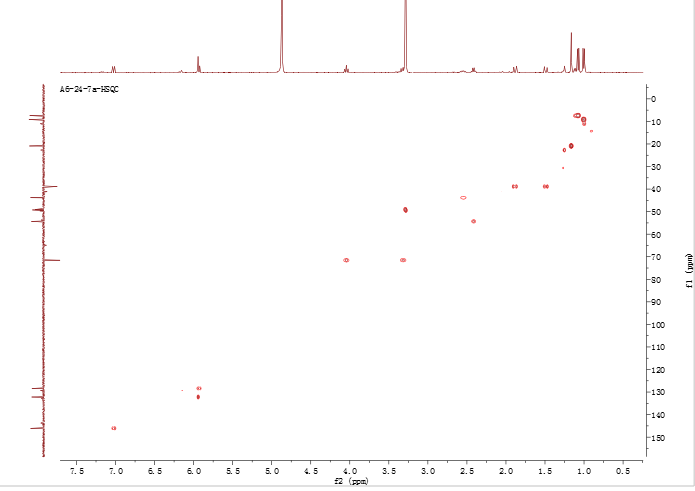


**Figure S11.** The HSQC spectrum of compound **2**.


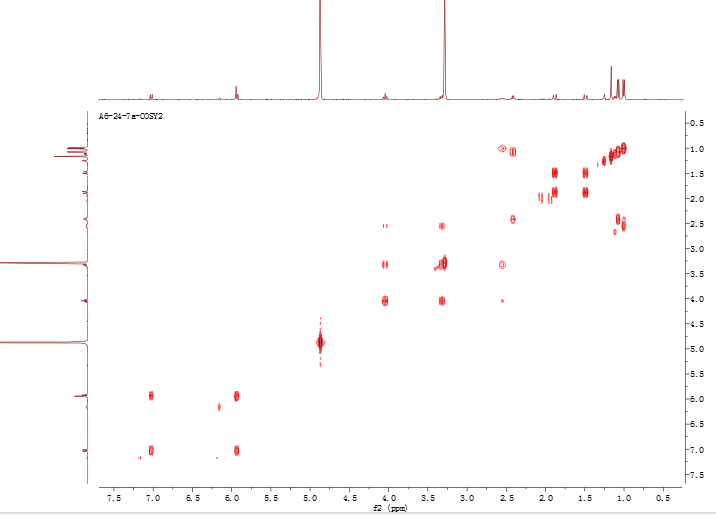


**Figure S12.** The ^1^H-^1^H COSY spectrum of compound **2**.


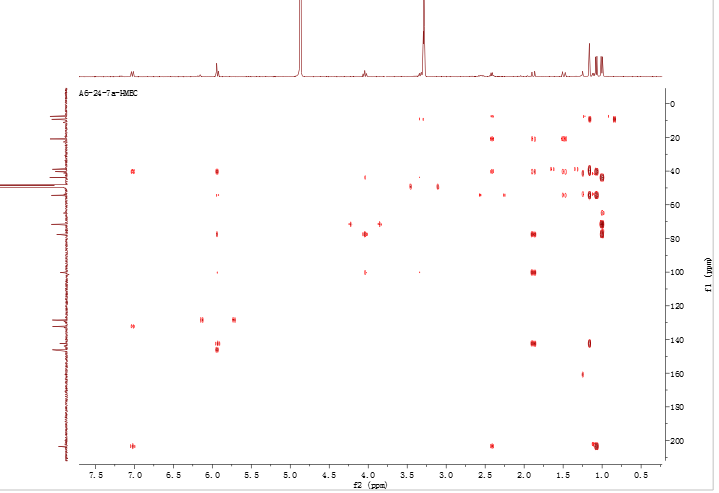


**Figure S13.** The HMBC spectrum of compound **2**.


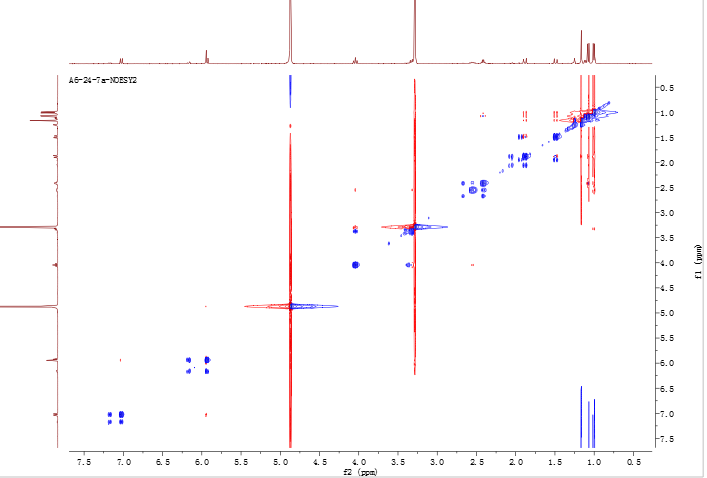


**Figure S14.** The NOESY spectrum of compound **2**.

**Figure S15.** The HRESIMS spectrum of compound **4**.


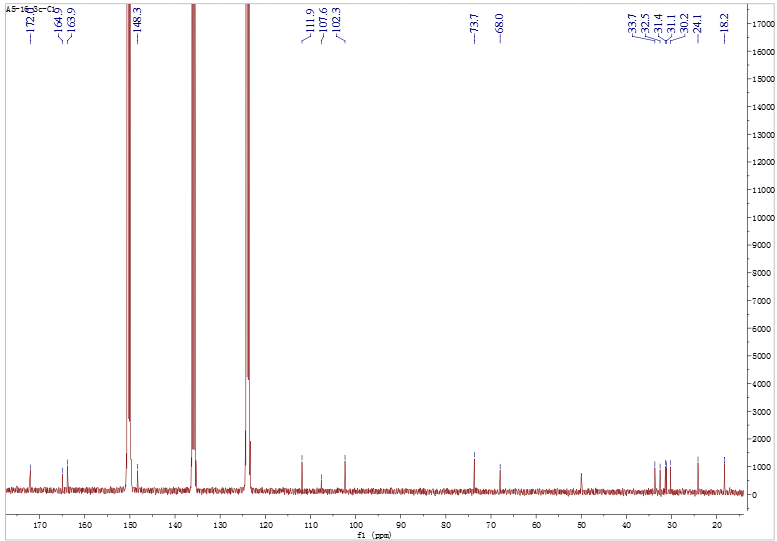


**Figure S16.** The ^13^C NMR spectrum of compound **4**.


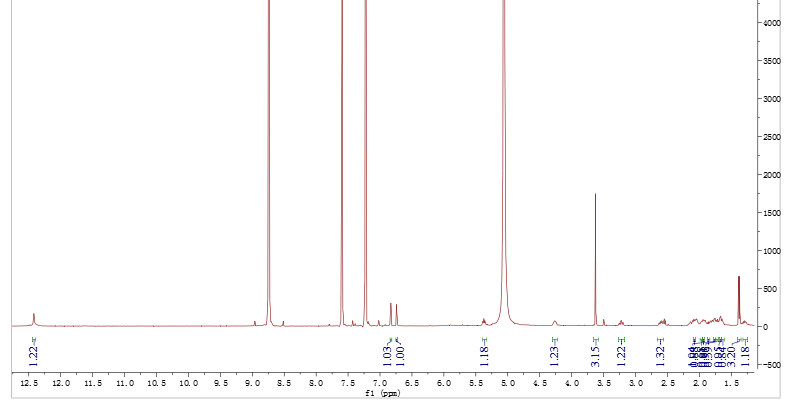


**Figure S17.** The ^1^H NMR spectrum of compound **4**.


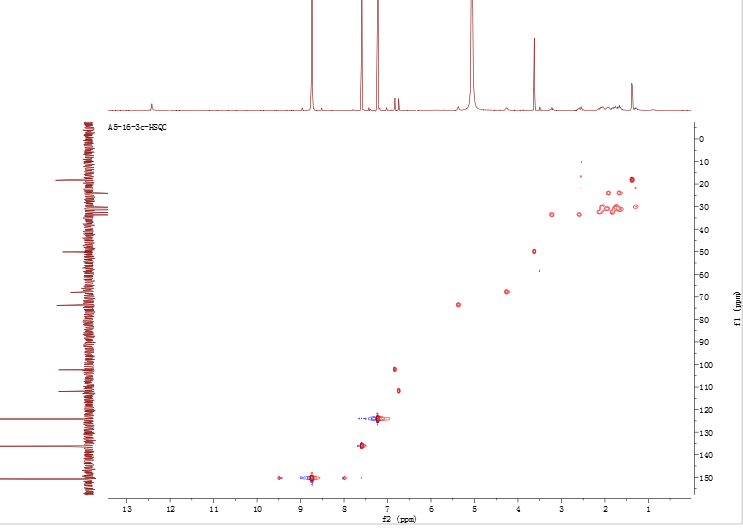


**Figure S18.** The HSQC spectrum of compound **4**.


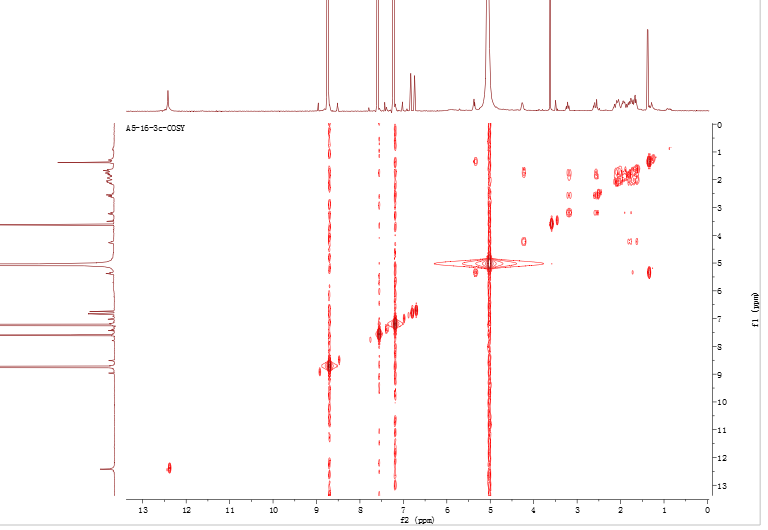


**Figure S19.** The ^1^H-^1^H COSY spectrum of compound **4**.


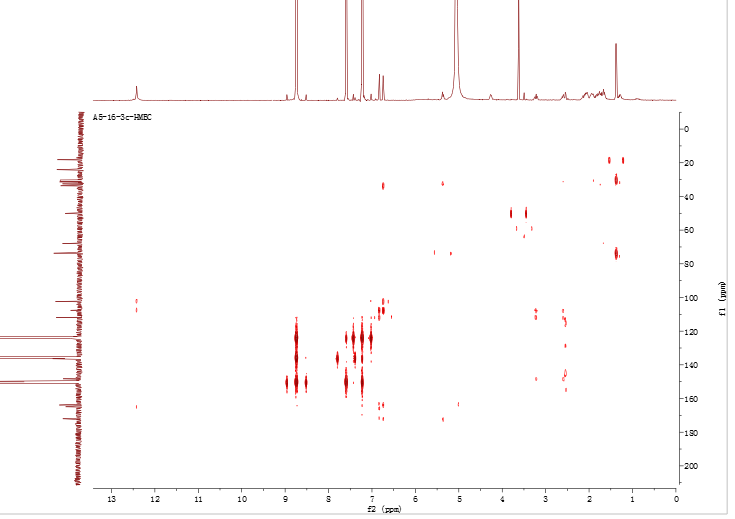


**Figure S20.** The HMBC spectrum of compound **4**.


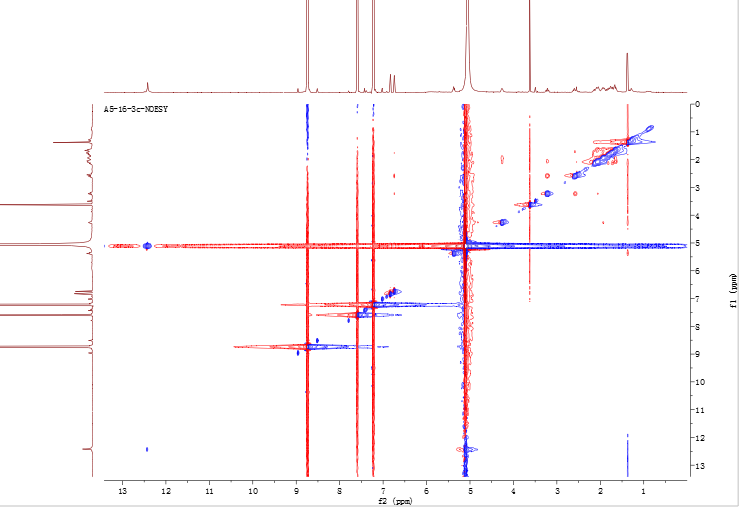


**Figure S21.** The NOESY spectrum of compound **4**.

**Figure S22.** The HRESIMS spectrum of compound **5**.


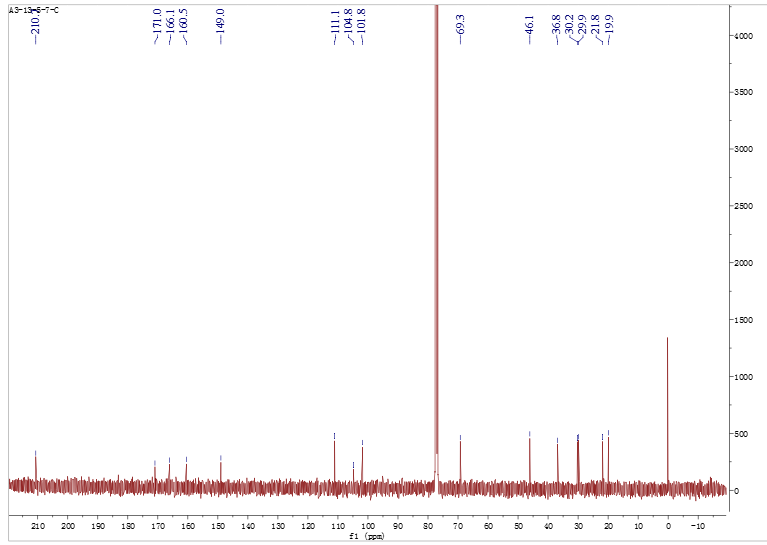


**Figure S23.** The ^13^C NMR spectrum of compound **5**.


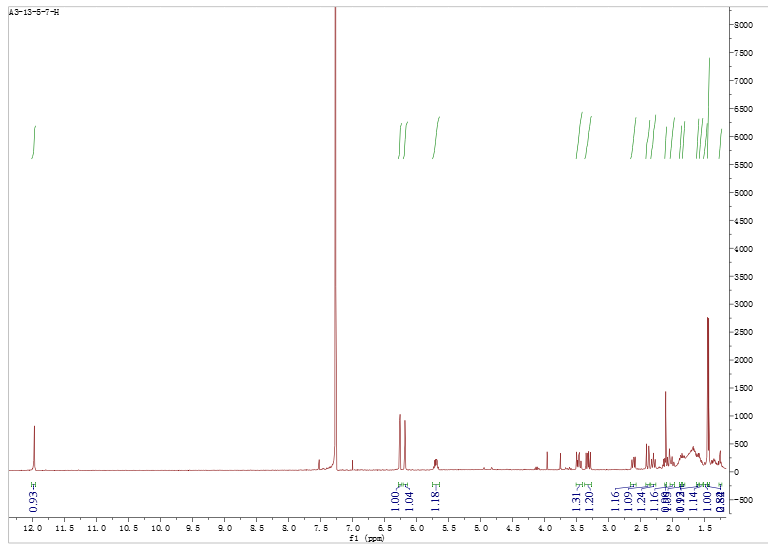


**Figure S24.** The ^1^H NMR spectrum of compound **5**.


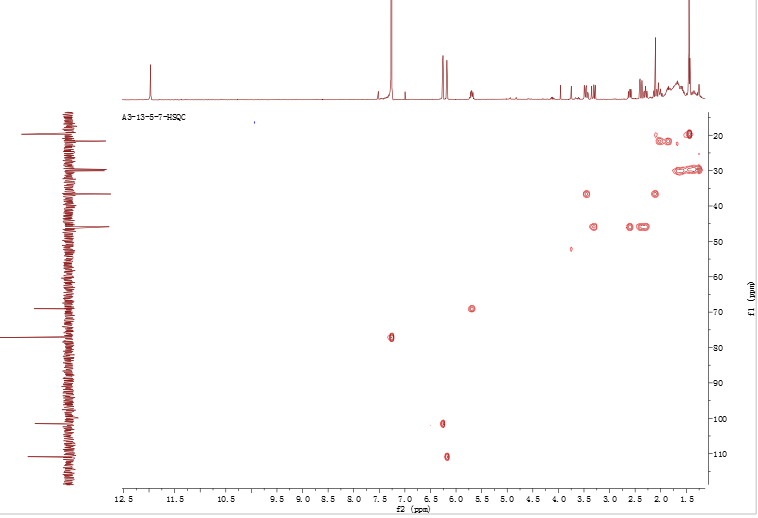


**Figure S25.** The HSQC spectrum of compound **5**.


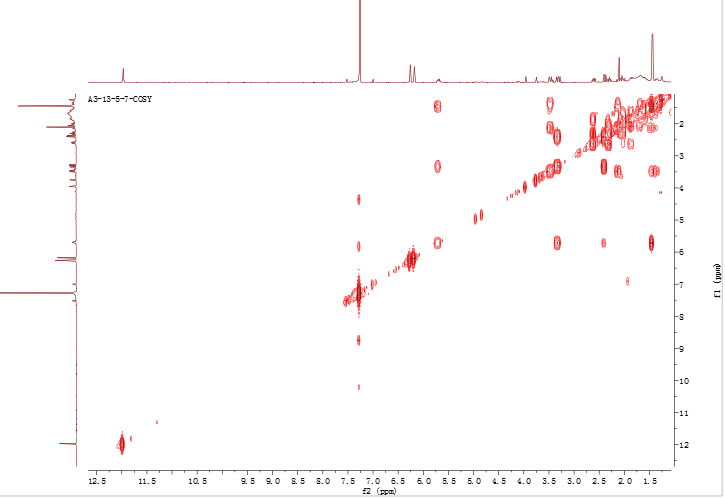


**Figure S26.** The ^1^H-^1^H COSY spectrum of compound **5**.


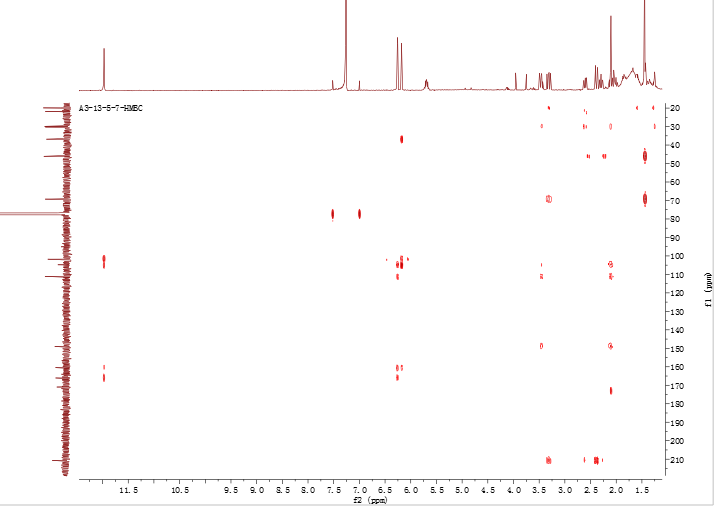


**Figure S27.** The HMBC spectrum of compound **5**.


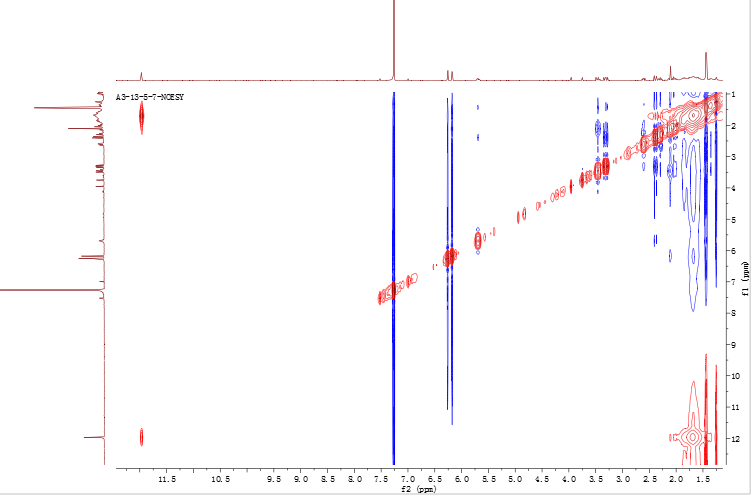


**Figure S28.** The NOESY spectrum of compound **5**.

**Figure S29.** The HRESIMS spectrum of compound **6**.


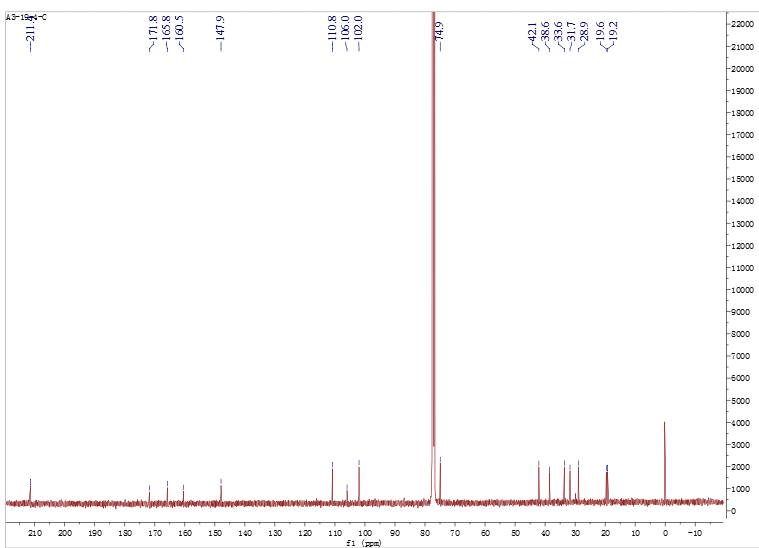


**Figure S30.** The ^13^C NMR spectrum of compound **6**.


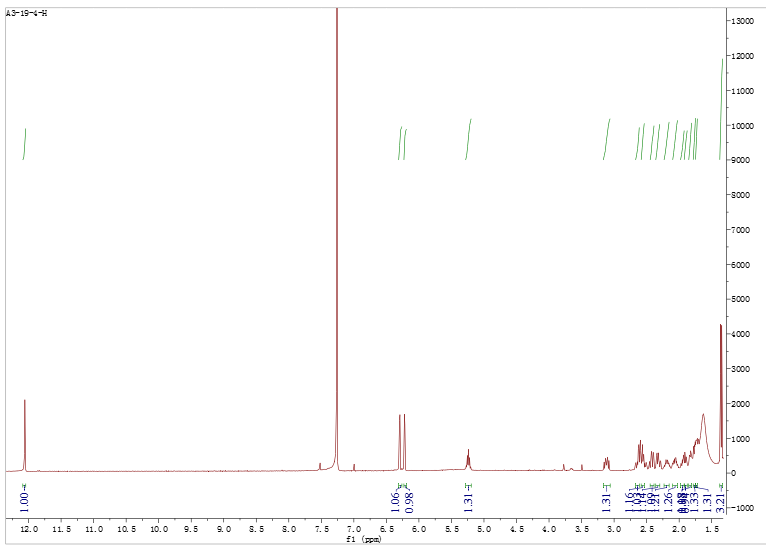


**Figure S31.** The ^1^H NMR spectrum of compound **6**.


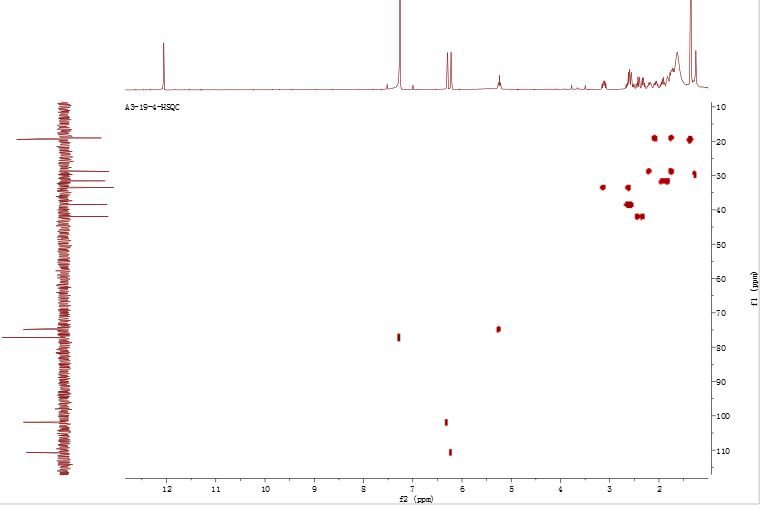


**Figure S32.** The HSQC spectrum of compound **6**.


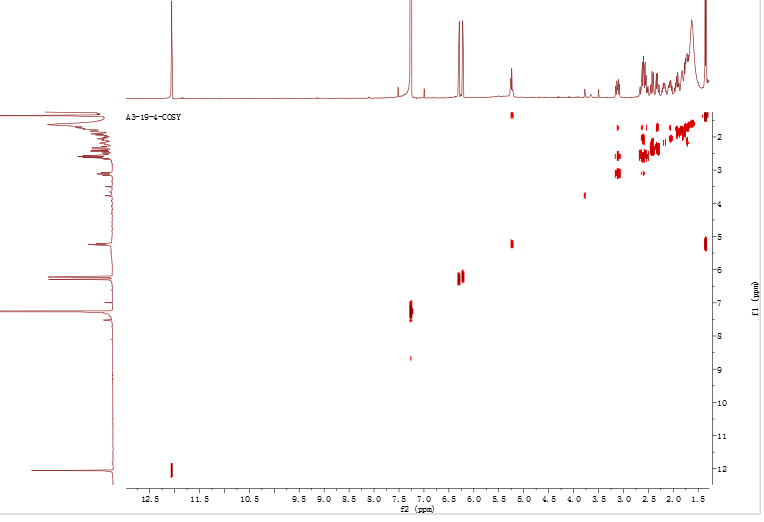


**Figure S33.** The ^1^H-^1^H COSY spectrum of compound **6**.


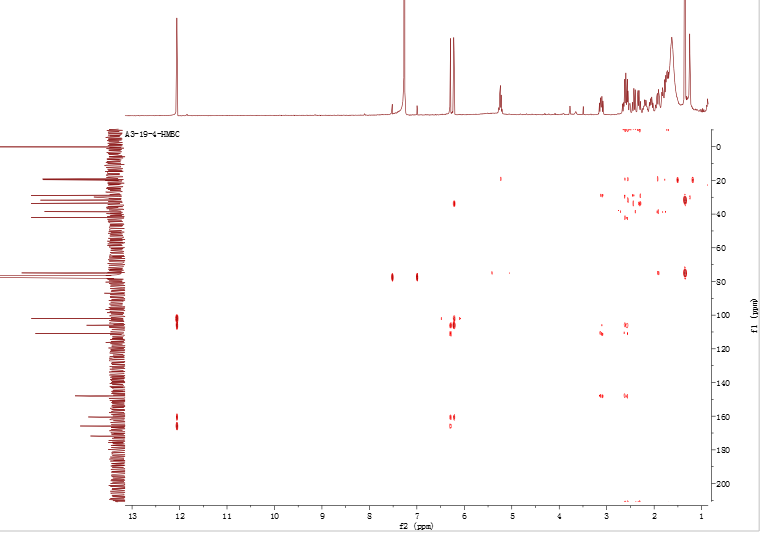


**Figure S34.** The HMBC spectrum of compound **6**.


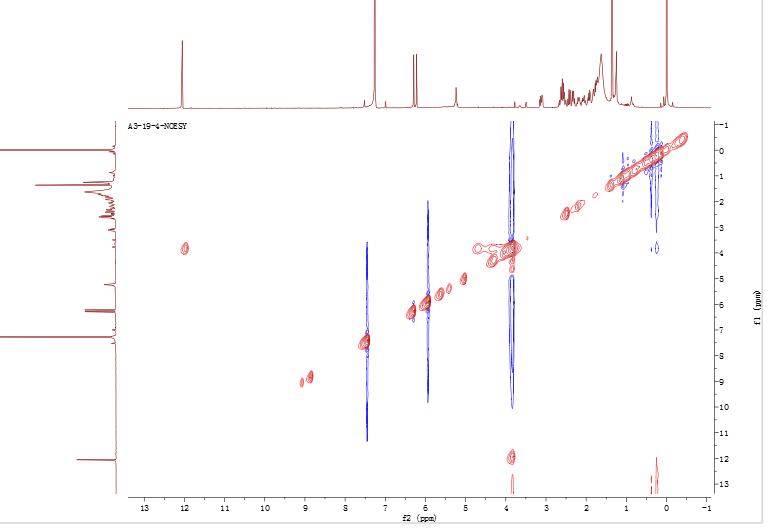


**Figure S35.** The NOESY spectrum of compound **6**.
